# Psychological support for an adolescent awaiting heart transplantation: A case report on psychological intervention using the Stanford Integrated Psychosocial Assessment for Transplant

**DOI:** 10.1002/pcn5.70087

**Published:** 2025-03-17

**Authors:** Kosuke Takano, Junko Tsutsui, Hidehiro Oshibuchi, Sayaka Kobayashi, Rie Akaho, Katsuji Nishimura

**Affiliations:** ^1^ Department of Psychology Meiji Gakuin University Minato‐ku Tokyo Japan; ^2^ Department of Psychiatry Tokyo Women's Medical University Shinjuku‐ku Tokyo Japan; ^3^ Department of Psychiatry, Saitama Medical Center Saitama Medical University Kawagoe‐shi Saitama Japan

**Keywords:** heart transplantation, pretransplant evaluation, psychological support, Stanford Integrated Psychosocial Assessment for Transplantation, ventricular assist device

## Abstract

**Background:**

Psychosocial factors significantly influence outcomes following heart transplantation (HT). In Japan, the prolonged waiting period for HT often requires a ventricular assist device (VAD), demanding strict adherence to self‐care regimens to prevent complications. The Stanford Integrated Psychosocial Assessment for Transplantation (SIPAT) identifies psychosocial barriers to successful transplantation. This case report describes the use of SIPAT‐guided psychological support for an adolescent awaiting HT.

**Case Presentation:**

A 16‐year‐old Japanese male with dilated cardiomyopathy underwent evaluation for HT. The SIPAT assessment revealed a total score of 25, with elevated scores in readiness for illness management (SIPAT A: 11) and social support (SIPAT B: 12), indicating challenges with understanding his condition and unstable family dynamics. Tailored interventions included psychotherapy and family psychoeducation. Following VAD implantation, initial nonadherence to self‐care prompted further evaluation using the Wechsler Adult Intelligence Scale‐Fourth Edition, which revealed deficits in processing speed and verbal comprehension. A transtheoretical model‐guided intervention was then implemented to improve self‐care behaviors. Periodic SIPAT assessments during routine check‐ups facilitated ongoing monitoring and timely interventions. Over 25 months, the SIPAT scores improved (14 and 15) and no adverse outcomes, such as infections, emergency hospitalizations, or nonadherence behaviors, were observed.

**Conclusion:**

This case highlights the effectiveness of early SIPAT‐guided psychological support and multidisciplinary interventions in addressing complex psychosocial issues in adolescents during the VAD waiting period. It underscores the critical need for regular psychosocial assessments and individualized care to optimize transplantation outcomes.

## Graphical abstract

We report the case of a 16‐year‐old patient who successfully managed a 25‐month heart transplantation (HT) waiting period. Despite the high risk of self‐care nonadherence during the prolonged HT waiting period, psychological support based on the Stanford Integrated Psychosocial Assessment for Transplantation was implemented, leading to a favorable course throughout the 25 months.

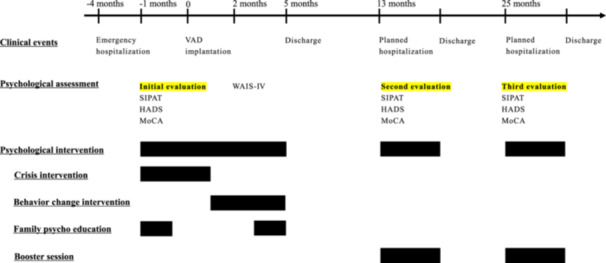

## BACKGROUND

Psychosocial factors prior to heart transplantation (HT) significantly influence posttransplant outcomes.^1^ In Japan, the waiting period for HT is prolonged, with the average waiting time for Status 1 patients reaching 1769 days.^2^ Most patients require implantation of a ventricular assist device (VAD) to maintain circulatory support during this waiting period. Consequently, these patients must adhere to strict self‐care regimens, which often necessitates limitations in daily activities and can negatively impact their quality of life.^3^ Nonadherence to VAD management and daily care regimens can lead to complications.^4^ Given the multifaceted nature of factors contributing to nonadherence in VAD patients,^5^ a comprehensive psychosocial assessment is essential.

The Stanford Integrated Psychosocial Assessment for Transplantation (SIPAT) was developed for the purpose of comprehensive psychosocial assessment,^6^ and we have developed a Japanese version (SIPAT‐J).^7^ The SIPAT was designed to standardize the psychosocial assessment of the transplant recipient and quantify transplant appropriateness.^6^ A critical aspect of utilizing the SIPAT is ensuring that assessments are conducted by a psychiatrist, clinical psychologist, or other healthcare professional with expertise in psychosocial evaluation. SIPAT scores in patients with left VAD have been shown to correlate with readmission outcomes.^8^ Importantly, SIPAT is not merely a tool to identify patients who meet exclusion criteria for HT. Comprehensive assessments using SIPAT, followed by tailored psychosocial support, are anticipated to play a crucial role in the care of patients awaiting HT.^9^

This paper presents the case of an adolescent patient awaiting HT. We discuss the psychological support provided during the pre‐VAD implantation period and throughout the transplant waiting period based on psychosocial assessments, including the SIPAT.

## CASE PRESENTATION

The patient was a 16‐year‐old male with no significant medical history. In X‐1, an abnormality was detected on an electrocardiogram during a routine medical checkup at the time of high school enrollment. Although a physician recommended a detailed examination, the patient did not seek medical attention due to a lack of subjective symptoms.

In January X, he was subsequently found to have cardiomegaly, congestion, and pleural effusion, leading to urgent hospitalization. He was diagnosed with dilated cardiomyopathy. As treatment with cardiotonic agents proved ineffective, he was transferred to our hospital for advanced care, including potential HT. Following his hospitalization, preparations for HT registration were initiated. As part of these preparations, a semistructured interview based on the SIPAT‐J was conducted by a psychiatrist and a clinical psychologist. The SIPAT‐J assesses 18 items, classified into four domains.^6^ Table 1 shows the psychosocial domains and factors measured by the SIPAT. The total SIPAT‐J score was 25, categorized as minimally acceptable. The high SIPAT A score reflected the patient's limited readiness, including insufficient knowledge of and motivation for transplantation. The SIPAT B score was elevated due to inadequate social support, particularly within his family and living environment. The SIPAT‐J results were disseminated to the multidisciplinary team, prompting the formulation of a care plan that prioritized the gradual provision of medical information and tailored psychological support. Psychotherapy facilitated by a clinical psychologist was initiated. During these sessions, the patient expressed confusion regarding the abrupt onset of his illness, difficulties navigating family relationships, and anxieties surrounding future treatment. A detailed exploration of family dynamics revealed challenges in decision‐making regarding VAD implantation. Initially, conflicting perspectives between the patient and his family created difficulties in reaching a consensus. To address these challenges, the clinical psychologist provided targeted family psychoeducation and participated in the informed consent process. The decision to proceed with VAD implantation was made collaboratively, with psychological interventions playing a crucial role in ensuring informed and shared decision‐making.

**Table 1:** Psychosocial domains and factors measured by the SIPAT.

*SIPAT A. Patient's Readiness Level and Illness Management (5 items)*
Item 1: Knowledge and understanding of medical illness process (that caused specific organ failure)
Item 2: Knowledge and understanding of the process of transplantation
Item 3: Willingness/desire for treatment (transplant)
Item 4: History of treatment adherence/compliance (pertinent to medical issues)
Item 5: Lifestyle factors (including diet, exercise, fluid restrictions, and habits, according to organ system)
*SIPAT B. Social Support System Level of Readiness (3 items)*
Item 6: Availability of social support system
Item 7: Functionality of social support system
Item 8: Appropriateness of physical living space and environment
*SIPAT C. Psychological Stability and Psychopathology (5 items)*
Item 9: Presence of psychopathology (other than personality disorders and organic psychopathology)
Item 10: History of organic psychopathology or neurocognitive impairment (i.e., illness or medication induced psychopathology)
Item 11: Influence of personality traits versus disorder
Item 12: Effect of truthfulness versus deceptive behavior
Item 13: Overall risk for psychopathology
*SIPAT D. Lifestyle and Effect of Substance Use (5 items)*
Item 14: Alcohol use, abuse, and dependence
Item 15: Alcohol abuse—risk for recidivism
Item 16: Illicit substance abuse and dependence
Item 17: Illicit substance abuse—risk for recidivism
Item 18: Nicotine use, abuse, and dependence

One month after VAD implantation, the patient was found to have difficulty acquiring self‐care behaviors. To assess his cognitive characteristics, the Wechsler Adult Intelligence Scale–Fourth Edition (WAIS‐IV) was administered. The WAIS‐IV results were reported to the physician and nurses, and based on these results, a multidisciplinary team initiated an intervention to help the patient acquire planned self‐care behaviors. The intervention was guided by the transtheoretical model.^10^, ^11^ The patient's readiness for behavior change was assessed and he was determined to be in the preparation stage. Psychotherapy sessions incorporated discussions on the impact of difficulties in acquiring self‐care behaviors on discharge timing and postdischarge life. Behavior change techniques were also employed, including sharing specific behaviors to be acquired, creating an action plan, monitoring progress with a behavior record sheet, and evaluating achievements. These interventions were designed to enhance the patient's self‐efficacy.

Five months after VAD implantation, the patient exhibited favorable physical recovery and successfully acquired the necessary self‐care skills. During the clinical psychologist's interventions, the patient underwent regular psychiatric evaluations. However, as the patient did not meet the diagnostic criteria for any psychiatric disorders, pharmacotherapy was not indicated. The clinical psychologist conducted regular SIPAT‐J assessments and booster sessions during the patient's planned check‐up hospitalizations. The SIPAT‐J scores improved over time, with a score of 14 (good candidate) recorded 13 months postsurgery and 15 (good candidate) recorded 25 months postsurgery. Self‐care behaviors were consistently maintained after discharge. Moreover, no adverse outcomes, such as infections, emergency hospitalizations, or nonadherence behaviors, were observed.

**Figure 1:**
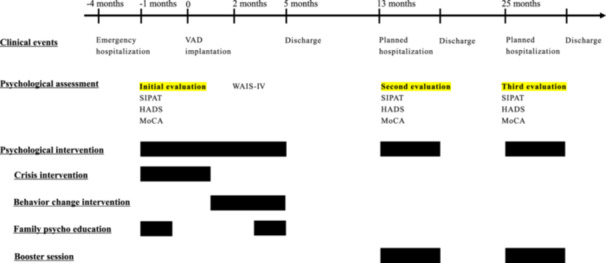
Clinical course of the present case. (Month): month is 0 for ventricular assist device (VAD) implantation. HADS, hospital anxiety and depression scale; MoCA, Montreal cognitive assessment; SIPAT, Stanford Integrated Psychosocial Assessment for Transplantation; WAIS‐Ⅳ, Wechsler adult intelligence scale–Fourth Edition.

The clinical course of this case is illustrated in Figure 1 and the psychological test results are summarized in Table 2.

**Table 2:** Psychological test results.

	Initial evaluation		Second evaluation	Third evaluation
Months: month is 0 for VAD implantation	−1	2	13	25
SIPAT				
Total	25	–	14	15
SIPAT A (patient's readiness level and illness management)	11	–	6	8
SIPAT B (social support system)	12	–	5	4
SIPAT C (psychological stability and psychopathology)	2	–	3	3
SIPAT D (lifestyle and effect lifestyle and effect of substance use)	0	–	0	0
HADS				
HADS‐A	7	–	5	3
HADS‐D	5	–	4	1
MoCA	25	–	21	21
WAIS‐Ⅳ				
Full scale	–	74	–	–
Verbal comprehension	–	77	–	–
Perceptual reasoning	–	84	–	–
Working memory	–	88	–	–
Processing speed	–	68	–	–

Abbreviations: HADS, hospital anxiety and depression scale; MoCA, Montreal cognitive assessment; SIPAT, The Stanford Integrated Psychosocial Assessment for Transplantation; VAD, ventricular assist device; WAIS‐Ⅳ, Wechsler adult intelligence scale–Fourth Edition.

## DISCUSSION

We report the case of a 16‐year‐old patient who successfully managed a 25‐month HT waiting period. Despite the high risk of self‐care nonadherence during the prolonged HT waiting period, psychological support based on the SIPAT was implemented, leading to a favorable course throughout the 25 months. To our knowledge, this case represents the first report of an adolescent patient successfully managing a VAD and awaiting HT for over 2 years with the aid of such support.

In this case, the initial SIPAT score was 25. Research indicates that recipients with a score of 21 or higher have significantly higher rates of missed appointments post‐HT and an increased likelihood of developing or experiencing a recurrence of mental illness.^12^ The SIPAT identified key issues, including confusion associated with the acute onset of illness, lack of understanding regarding heart disease and transplantation, and instability of social support. Previous studies have demonstrated an association between psychosocial factors prior to VAD implantation and subsequent negative outcomes.^8^, ^13^, ^14^ Early implementation of the SIPAT offers the advantage of identifying specific psychosocial factors that may contribute to negative outcomes, enabling targeted interventions. In this case, marked by adolescent onset and complex psychosocial challenges, initial interventions included crisis management to address confusion related to the acute onset of illness and the patient's lack of understanding of heart disease and transplantation. Furthermore, due to the observed instability of social support, particularly the lack of family support, family psycho education was initiated by a multidisciplinary team at an early stage. These efforts likely contributed to establishing and maintaining robust social support. In cases with complex backgrounds, early identification of problems and the provision of highly individualized, multidisciplinary support informed by the SIPAT may improve outcomes.

Furthermore, as the patient experienced difficulty acquiring self‐care behaviors, the WAIS‐IV was administered alongside the SIPAT to assess his cognitive abilities. The WAIS‐IV proved valuable in analyzing the patient's detailed cognitive characteristics and developing an intervention plan to facilitate self‐care behavior acquisition. Support strategies that incorporated the patient's cognitive characteristics, as revealed by the WAIS‐IV, were instrumental in promoting the successful acquisition and maintenance of effective self‐care behaviors. Additionally, a behavioral change intervention based on the transtheoretical model was implemented.^9^, ^10^ Assessing the patient's readiness for behavior change and tailoring interventions to match his readiness level were pivotal in achieving self‐care behavior acquisition. Providing feedback on the WAIS‐IV results to the patient and offering support designed to enhance self‐efficacy were also critical elements of the intervention. These approaches collectively contributed to the positive outcomes observed in this case.

While many studies have investigated the association between pretransplant psychosocial factors and posttransplant outcomes using the SIPAT,^12^, ^15^, ^16^, ^17^ fewer have examined its utility during the transplant waiting period. This period is often prolonged for HT candidates in Japan, who typically receive VAD as a bridge to transplantation.^2^ In such cases, repeated SIPAT assessments are crucial to monitor evolving psychosocial needs and adjust intervention strategies accordingly. This case study demonstrates the value of periodic SIPAT evaluations in facilitating early problem detection and timely intervention, both of which are critical for effectively managing the challenges of extended transplant waiting periods.

This case report has two limitations. First, the follow‐up period was limited. The waiting period for HT is ongoing and posttransplant outcomes could not be assessed. Second, the SIPAT was originally developed as a psychosocial assessment tool for adults.^6^ Previous studies on pre‐transplant psychosocial assessment in pediatric patients recommend evaluating factors that align with those included in the SIPAT.^18^, ^19^ These findings support the relevance of SIPAT‐related domains in assessing psychosocial readiness for transplantation in younger patients. Although its usefulness was demonstrated in this adolescent case, research on the applicability of SIPAT for adolescent recipients remains limited. Future efforts should focus on developing psychosocial assessment tools specifically designed for adolescent and pediatric transplant candidates.

## CONCLUSION

This case report demonstrates the successful management of a 25‐month HT waiting period in an adolescent patient at high risk for self‐care nonadherence through psychological support based on the SIPAT. The case underscores the potential benefits of early SIPAT assessment and individualized psychosocial support in achieving favorable outcomes.

## AUTHOR CONTRIBUTION

Kosuke Takano and Rie Akaho provided treatment and care for the patients, and Kosuke Takano drafted the manuscript. Junko Tsutsui, Hidehiro Oshibuchi, and Sayaka Kobayashi contributed to the study design and critically reviewed and revised the draft. Hidehiro Oshibuchi contributed to the acquisition of funding. Katsuji Nishimura provided overall support for the study and managed this project. All authors approved the final version of the manuscript.

## CONFLICT OF INTEREST STATEMENT

Katsuji Nishimura is a Field Editor of *Psychiatry and Clinical Neurosciences Reports* and a co‐author of this article. To minimize bias, they were excluded from all editorial decision‐making related to the acceptance of this article for publication.

## ETHICS APPROVAL STATEMENT

N/A.

## PATIENT CONSENT STATEMENT

Written informed consent for presentation of his clinical course was given by the patient and his parent.

## CLINICAL TRIAL REGISTRATION

N/A.

## Data Availability

The data for this study are not publicly available due to privacy and ethical restrictions.
